# Supplementary material for: Increasing Protein Content of Rice Flour with Maintained Processability by Using Granular Starch Hydrolyzing Enzyme
Source: Molecules. 2023 Apr 17;28(8):3522. doi: 10.3390/molecules28083522 (PMC10141220; doi:10.3390/molecules28083522)
Supplement: Supplementary file 1 [file molecules-28-03522-s001.zip › molecules-2275342-supplementary.pdf]

Increasing protein content of rice flour with maintained processability of starch by using granular starch hydrolyzing enzyme

Jinxing Zhai[1], Xiaoxiao Li[1]*, Birte Svensson[2], Zhengyu Jin[1,3,4,5], Yuxiang Bai[1,3,4,5]*

[1] State Key Laboratory of Food Science and Technology, Jiangnan University, Wuxi, 214122, China;

[2] Enzyme and Protein Chemistry, Department of Biotechnology and Biomedicine, Technical University of Denmark, DK-2800 Kongens Lyngby, Denmark

[3] School of Food Science and Technology, Jiangnan University, Wuxi, 214122, China;

[4] Collaborative Innovation Center of Food Safety and Quality Control in Jiangsu Province, Jiangnan University, Wuxi, 214122, China;

[5] International Joint Laboratory on Food Safety, Jiangnan University, Wuxi, 214122, China;

*Corresponding author at: State Key Laboratory of Food Science and Technology, Jiangnan University, Wuxi, 214122, China.

E-mail address:

xxiaoli@jiangnan.edu.cn

ybai@jiangnan.edu.cn

Tel number: +86-0510-85329291

Table S1. The DSC parameters [1] of native and partially hydrolyzed RF (the second endothermic peak).

Samples [2]	T_{o2} (°C)	T_{p2} (°C)	T_{c2} (°C)	T_{c2}-T_{o2} (°C)	ΔH_2 (mJ/mg)
RF-N	79.63±0.21 ^{a}	81.87±0.44 ^{a}	84.41±0.22 ^{a}	4.78±0.23 ^{a}	0.94±0.12 ^{a}
RF-1	78.35±0.20 ^{b}	79.93±0.22 ^{b}	82.69±0.16 ^{c}	4.34±0.29 ^{a}	1.24±0.19 ^{a}
RF-6	78.57±0.31 ^{b}	79.87±0.22 ^{b}	82.99±0.25 ^{bc}	4.42±0.11^{a}	1.25±0.13 ^{a}
RF-24	79.06±0.10 ^{ab}	80.24±0.14 ^{b}	83.53±0.21 ^{b}	4.47±0.28 ^{a}	1.03±0.16 ^{a}

[1] Means ± SD values followed by different lowercase letters of the RF group are significantly different ($p < 0.05$). T_o: Onset temperature; T_p: peak temperature; T_c: conclusion temperature; ΔH: enthalpy change. “2” represents the second endothermic peak in the RF group. [2] RF represents rice flour. Numbers refer to the hydrolysis time (hours). RF-N is native rice flour.

Table S2. Composition of oligosaccharides [1] released during RS hydrolyzed by GSHE

Samples[2]	The composition of oligosaccharides（%）				
	G1	G2	G3	G4	G5
RS-1	24.91±0.35^{E}	62.37±0.47^{A}	1.47±0.26^{A}	4.42±0.51^{D}	6.82±0.31^{A}
RS-3	31.47±1.45^{D}	55.25±1.87^{B}	1.21±0.18^{AB}	6.22±0.52^{C}	5.94±0.12^{B}
RS-6	35.21±0.47^{C}	51.88±0.14^{C}	1.03±0.06^{B}	6.73±0.02^{C}	5.53±0.06^{C}
RS-9	38.25±0.47^{B}	49.91±0.07^{D}	0.17±0.04^{C}	7.26±0.06^{B}	4.96±0.13^{D}
RS-24	48.68±0.91^{A}	38.82±0.35^{E}	0.06±0.01^{D}	8.19±0.04^{A}	3.87±0.16^{E}

[1] Means ± SD values followed by different uppercase letters of the RS group are significantly different ($p < 0.05$). G1-G5 mean the glucose, maltose, maltotriose, maltotetraose, and maltopentaose, respectively. [2] RS represents rice starch, and numbers refer to the hydrolysis time (hours).
